# Frailty and Age Impact Immune Responses to Moderna COVID-19 mRNA Vaccine

**DOI:** 10.21203/rs.3.rs-1883093/v1

**Published:** 2022-08-01

**Authors:** Charles T. Semelka, Michael E. DeWitt, Maria W. Blevins, Beth C. Holbrook, John W. Sanders, Martha A. Alexander-Miller

**Affiliations:** Wake Forest University School of Medicine; Wake Forest University School of Medicine; Wake Forest University School of Medicine; Wake Forest University School of Medicine; Wake Forest University School of Medicine; Wake Forest University School of Medicine

**Keywords:** Frailty, Immune Function, COVID-19

## Abstract

**BACKGROUND:**

Immune responses to COVID-19 mRNA vaccines have not been well characterized in frail older adults. We postulated that frailty is associated with impaired antibody and cellular mRNA vaccine responses.

**METHODS:**

We followed older adults in a retirement facility with longitudinal clinical and serological samples from the first Moderna mRNA-1273 vaccine dose starting in February 2021 through their 3rd (booster) vaccine dose. Outcomes were antibody titers, antibody avidity, and AIM+ T cell function and phenotype. Statistical analysis used antibody titers in linear mixed-effects linear regression with clinical predictors including, age, sex, prior infection status, and clinical frailty scale (CFS) score. T cell function analysis used clinical predictors and cellular phenotype variables in linear regression models.

**RESULTS:**

Participants (n=15) had median age of 90 years and mild, moderate, or severe frailty scores (n=3, 7, or 5 respectively). After 2 vaccine doses, anti-spike antibody titers were higher in 5-fold higher in individuals with mild frailty compared to severe frailty and 9-fold higher in individuals with prior COVID-19 infection compared to uninfected (p=0.02 and p<0.001). Following the booster, titers improved regardless of COVID-19 infection or frailty. Antibody avidity significantly declined following 2 vaccine doses regardless of frailty status, but reached maximal avidity after the booster. Spike-specific CD4+ T cell responses were modulated by frailty and terminally differentiated effector memory TEMRA cells, and spike-specific TFH cell responses were inversely correlated with age. Additionally, an immune-senescent memory T cell phenotype was correlated with frailty and functional decline.

**CONCLUSIONS:**

We described the separate influences of frailty and age on adaptive immune responses to the Moderna COVID-19 mRNA vaccine. Though overall antibody responses were robust, higher frailty diminished initial antibody quantity, and all older adults had impaired antibody avidity. Following the booster, antibody responses improved, overcoming the effects of age and frailty. CD4+ T cell responses were independently impacted by age, frailty, and burden of immune-senescence. Frailty was correlated with increased burden of immune-senescence, suggesting an immune-mediated mechanism for physiological decline.

## Introduction

The COVID-19 pandemic has disproportionately affected the population of nursing home residents, accounting for approximately 25% of the US COVID-19 related deaths, despite making up only 5% of the population of older adults.^1–4^ There is a high rate of COVID-19 mRNA vaccination in nursing homes, with over 80% of residents having received a booster dose.^3,4^ However, evidence describes waning antibody levels and vaccine effectiveness in older adults compared to young and middle-aged adults.^5–9^ Frailty has been correlated with decreased effectiveness of influenza, varicella-zoster, and pneumococcal pneumonia vaccines.^10–12^ There is emerging evidence for impaired COVID-19 vaccine responses in community-dwelling frail older adults, but evaluation of immune function was limited.^13,14^ While COVID-19 vaccine immune responses have been studied in the nursing home setting,^15–18^ the impact of frailty on vaccine responsiveness has not been adequately assessed due to imprecise/lack of measurement of frailty,^18–24^ homogeneous frailty status of populations,^25^ or limited immunological assessments.^26^

Frailty is a geriatric syndrome leading to worsened health outcomes due to impaired regulation of homeostasis, and it serves as a marker of biological aging.^27–30^ This is a common condition with 25–50% of community-dwelling individuals are frail, and it is associated with impaired function which portends a higher odds of nursing home placement.^31,32^ Frailty is also a reliable predictor for adverse health outcomes following COVID-19 infection.^33,34^ Measurement of frailty can be accomplished through clinical assessment of physical and functional status or through use of a frailty index to quantify accumulation of health deficits.^30,35,36^ The clinical frailty scale (CFS) is a quantitative frailty measure based upon comprehensive geriatric clinical assessment, and it has been well-validated in COVID-19 research in nursing home populations.^34,35,37^

Vaccination is a cost-effective and practical public health measure in the aging population for whom infections remain a leading cause of morbidity, mortality, and impaired quality of life.^38^ However, vaccine responsiveness is impaired by changes associated with the aging immune system, termed immune-senescence. These changes have impacts across the immune system, including impaired germinal center responses and increased inflammatory subsets of aging B cells,^39^ and losses of the highly-proliferative naïve cell reservoir and predominance of memory populations specific to latent infections in aging T cells.^40–42^ Immune-senescent changes have been associated with impaired antibody and cellular vaccine responses in older adults.^43–46^ Furthermore, immune-senescence is characterized by inflammation and impaired tissue repair mechanisms that lead to disease pathogenesis, informing a model where immune dysfunction contributes to a frail state.^47–51^ Yet, the associations between immune-senescence and frailty in vaccine responsiveness remain poorly defined.

Antibody seroconversion is one of the main measures of vaccine responsiveness. Antibodies are produced by B cells and target specific epitopes on pathogens, which confers protection across variants.^18,21,52,53^ Higher quantity antibody titers detected with ELISA are associated with protection from adverse health-related outcomes from COVID-19 and influenza,^54–56^ but frailty and older age have been associated with waning of COVID-19 vaccine-elicited antibody.^7,13,19,25,57^ However, quantitative antibody assays are incomplete descriptors of immunity to SARS-CoV-2.^58^ A surrogate of protection from infection is antibody neutralization, which commonly targets the SARS-CoV-2 spike protein receptor binding domain (RBD).^59,60^ Avidity is another critical parameter of antibody function, which measures the effectiveness of antibody binding.^61,62^ Avidity assays can be performed over the vaccine response time course to describe the maturation of antibody, as B cells undergo selection in the germinal center.^61,63^

T cells provide long-lasting immunity to conserved SARS-CoV-2 epitopes, which confers protection from severe disease across variants.^64–67^ A practical and highly sensitive approach for measuring T cell responses to SARS-CoV-2 used activation induced markers (AIM), and this assay has been used to describe a complex relationship between COVID-19 infection and cellular responses in older adults.^68^ Impaired T cell responses were described in older adults, including a paucity of antigen recognition following COVID-19 infection, which is postulated as the mechanism of severe health outcomes associated with COVID-19 disease.^69–71^ Older individuals who survive COVID-19 infection develop improved cellular memory responses following vaccination compared with infection-naïve individuals.^25,72^ The cellular immune profile associated with impaired vaccine responses in older adults has been described for influenza vaccines, which includes alterations in CD8 + memory and CD4 + T follicular helper cells (T_FH_). However, these responses have yet to be explored in frail individuals following COVID-19 vaccination.

While there is evidence for impaired antibody and cellular COVID-19 vaccine responses in older adults, the impact of biological age and frailty remains unclear.^25,73^ Furthermore, the underlying mechanisms which impact vaccine responses in frail individuals remains poorly understood.^13,14^ Here we tested the hypothesis that frailty in older adults in a retirement community will correlate with decreased vaccine responsiveness. We assessed responses to the Moderna mRNA COVID-19 vaccine by measuring the quantity and quality of vaccine-elicited antibody, as well as vaccine-specific T cell responses.

## Methods

### Study Design

Wake Forest University School of Medicine IRB authorized the study and consent forms under IRB#71181. Participants were recruited from a single retirement facility staffed by academic geriatricians. All retirement facility residents were eligible for participation. Exclusion criteria included lacking capacity to consent, and clinically significant changes in health status including vital sign instability and/or unstable health conditions. Older adults living in either a nursing home or assisted living were assessed by a physician member of the research team for the capacity to consent at study enrollment prior to volunteering written informed consent. Capacity to participate was assessed at each subsequent sample collection.

A cohort of older adults in a retirement facility were followed from the first Moderna mRNA-1273 vaccine dose in February 2021 with blood sample collections at baseline, second dose (4 weeks post first dose), and 2 weeks, 3 months, and 6 months post second dose. A subset of participants was followed for a final blood collection 2 weeks following a third Moderna mRNA-1273 vaccine dose (booster dose). Each study visit blood draw occurred in participant domiciles, which was accompanied by a comprehensive geriatrics clinical assessment with frailty status characterized using the 9-point Clinical Frailty Scale developed by Rockwood.^35^ Health conditions, including prior COVID-19 infection, were verified through review of participants’ medical information in the electronic health record (EHR) [EPIC Systems, Madison, WI USA]. For our primary outcomes we measured antibody titers, avidity, and cellular activation to the first 2 COVID-19 mRNA vaccine doses. For our secondary outcomes we measured antibody titers and avidity to the booster vaccine dose. We conducted exploratory analysis of the association cellular immune phenotype with participant frailty status.

### Human Sample Collection and Storage

Sample collections and timeframe were modeled from the Moderna BNT162b2 vaccine study.^55^ Plasma was collected using Heparin vacutainer tubes (BD Biosciences). Peripheral blood mononuclear cells (PBMC) were separated from fresh plasma using Ficoll (Cytiva) density gradient centrifugation in Leucosep tubes (Greiner Bio-One) and were cryopreserved in 10% dimethylsulfoxide (DMSO from Sigma) supplemented with fetal bovine serum (FBS from Atlanta Biologicals). Serum was drawn into SST vacutainer tubes containing clotting activator (BD Biosciences) and left at room temperature for 30–60 min, before centrifuging for 10 min at room temperature. Serum and plasma following PBMC separation were aliquoted and frozen at − 80°C.

### Assessment of Humoral Responses

## Elisa

We performed enzyme-linked immunosorbent assays (ELISA) to quantify anti-spike and anti-RBD IgG antibodies from serum and plasma with previously established assays.^74^ Antibodies were validated by the manufacturer and titrated for ELISA by serial dilution.

Reagents included phosphate buffered saline (PBS from BioWhittaker, Lonza ), Tween 20 (Fisher), TMB for chromogenic development (Sigma-Aldrich), and milk (BD Sciences). Ninety-six half well-plates (Greiner Bio-One) were coated with antigen or PBS for a negative control overnight at 4°C, then washed with PBS-0.1% Tween and blocked with PBS-3% milk at room temperature for 1 h. Antigens used in ELISA were SARS-CoV-2 Washington-1 spike protein at 12.5 ng/ml or RBD protein at 25 ng/ml (BEI Resources, NIAID, NIH). Aliquoted serum samples were titrated in eight 2-fold serial dilutions in PBS-1% milk starting at a minimum dilution of 1:100, and given high titers in some subjects, starting dilution was increased to a maximum of 1:12,800. Plates were incubated with diluted serum for 2 h at room temperature, washed with PBS-0.1% Tween and incubated with goat anti-Human IgG HRP (1:4,000) (Southern Biotech) detection antibody in PBS-0.1% milk for 1 h at room temperature. Plates were washed with PBS-0.1% Tween and developed with TMB for 30 minutes at room temperature in the dark. The reaction was stopped with 2N H_2_SO_4_ and the plates were read at 450 nm immediately after stopping. The limit of detection was defined as 1:100.

### Avidity

Quality of antibody binding was assessed with an avidity assay following a previously established procedure.^75,76^ The ELISA assay was performed with spike protein as described above, with modifications as follows. Participant serum was used at a dilution that resulted in half-maximal peak ELISA titer values. Prior to incubation with detection antibody, sodium thiocyanate (NaSCN from Acros Organics) was added at a starting concentration of 5 M with 2-fold dilutions to 0.195 M in PBS per well. PBS alone was added to a negative control well. After a 15 min incubation at ambient temperature, the plate was washed again with PBS-Tween, detection antibody was added, and the ELISA was continued as previously described. The calculation of the avidity index will be described in more detail in the statistical analysis subsection.

### Stimulation and Staining of Human Peripheral Blood Mononuclear Cells

Peptides from Washington-1 SARS-CoV-2 spike protein were obtained from BEI Resources (NR-52402). The entire 181 individual peptides were combined in a peptide mega-pool. Each peptide was resuspended in 70% acetonitrile, pooled, aliquoted, and lyophilized before storing at −80 C.

PBMC samples were thawed, washed, and resuspended in RPMI media with 5% human albumin and L-Glutamine with between 5×10^5^ to 1×10^6^ cells per well in 96-well U bottom plates. Cells were cultured for 24 hours in the presence of SARS-CoV-2 spike pooled peptides [0.8 μg/ml of each peptide] or 10 μg/mL PHA (Sigma) at 37°C in a humidified atmosphere containing 5% CO2. Incubation with an equimolar amount of DMSO (Sigma) was performed as negative control.

Surface staining was performed on PBMCs following 24h stimulation culture. Cells were resuspended in 100 ml PBS with 2% FBS (FACS buffer), then underwent wash and centrifugation between steps, including Live/Dead stain (Biolegend), Fc block (Biolegend), and antibody cocktail stain (antibodies from Biolegend) for 30 minutes at 4°C in the dark. Following surface staining, cells were washed twice with FACS buffer. After the final wash, cells were resuspended in 200ul PFA fixation buffer.

### Flow Cytometric Activation Induced Cell Marker (AIM) Assay

We used activation induced surface markers (AIM), a cytokine independent approach, for functional measurement of T cell activation. This technique has previously been reported to be highly sensitive for detection of rare cell populations including circulating Tfh cells.^77^ We defined a 13-color flow cytometry panel of lymphocyte lineage markers (Supplementary Table 1) to assess AIM and the adaptive cellular immune-phenotype of vaccine respondents.

All samples were acquired on a Fortessa X20 cytometer configured with five excitation lasers (355, 405, 488, 561, 640 nm) and 20 detectable parameters (BD Biosciences, San Diego, CA). Data in .fcs format were exported from the FACSDIVA software of the cytometer and processed directly using FlowJo (version 10.1, FlowJo, LLC).

## Statistical Analysis

Data was analyzed using R, version 4.0.3 (R Foundation, Vienna Austria). Figures were created using Prism 9.1.0 (GraphPad Software, San Diego California). A two-sided significance of P < 0.05 was considered significant. Results from Bayes statistics yielded a posterior probability distribution (PD), which was converted to a P value for general interpretability.

Using linear mixed regression models we examined the association of log-transformed antibody titers with fixed effects including clinical and demographic variables (age, sex, frailty status, and prior COVID-19 infection) for the secondary vaccine dose response with each study participant representing a random intercept. For the analysis of the response to the booster vaccine dose we used Bayesian linear mixed models to avoid singular model fits. Uninformative (flat) priors were used for all coefficients and intercept. Avidity assays used Prism software to calculate IC50 values for inhibitor response curves to estimate the concentration of inhibitor required for half-maximal antibody binding.

Cellular analysis used linear regression models to report results of AIM + T cells following stimulation with spike pooled peptides with subtraction of DMSO negative control values. We analyzed the relationship of antibody and cellular response with geometric mean titers (GMT) of individuals’ antibody titers following 2 vaccine doses in association with AIM + T cells. Next, we reported the associations of clinical variables and the cellular immune-phenotype with AIM + T cells in multivariable models using stepwise leap forward selection techniques from the Caret R package. Parameters were selected using 10-fold cross validation to minimize overall model root mean squared error. Final models were fit using predictors with the highest explanatory power measured by the lowest RMSE. Additionally, we conducted exploratory analysis with correlation plots to describe the associations of participant frailty status with their immune-phenotype.

## Results

In a cohort of 15 participants living in retirement facility, median (IQR) age was 90 years (84, 96). Twelve participants were female (80%) and 3 individuals (20%) had COVID-19 infection prior to vaccination. Participant frailty status was characterized using the CFS frailty scale based on geriatric assessment with a range of mild frailty (n = 3), moderate frailty (n = 7), and severe frailty (n = 5) [Table 1]. Fourteen individuals were followed to 6 months post the second vaccine dose. Eight individuals had blood collected 2 weeks after the third vaccine (booster) dose. Censoring occurred due to death, loss to follow up, and decline in clinical status. One individual developed COVID-19 infection after the 6-month post second dose but prior to booster dose.

### Antibody Responses

**Figure 1:**
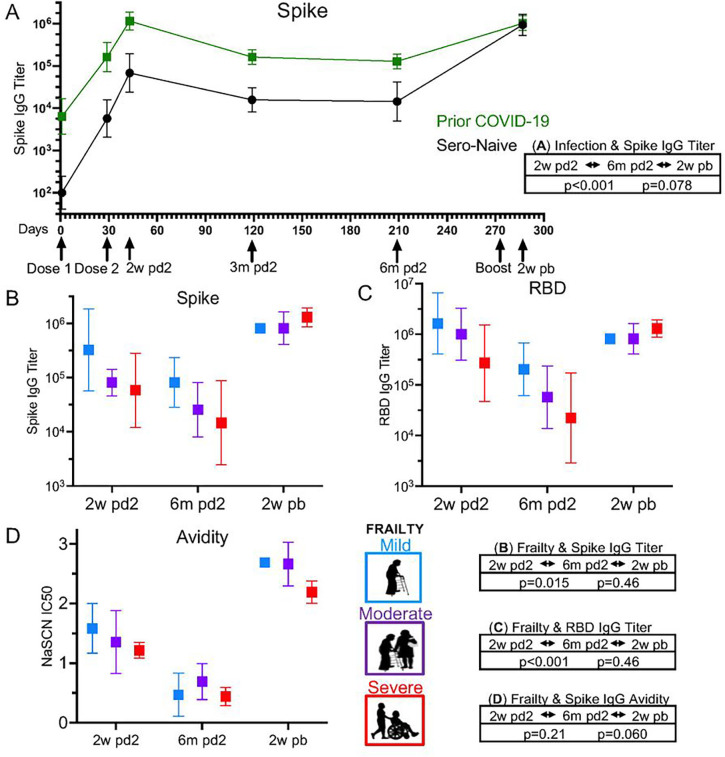
Frailty Impacts Antibody Response in Older Adults. ELISA was performed on patient samples for antibodies to spike IgG and RBD IgG over the study time course: Dose 1 (baseline), Dose 2 (28 days), 2-weeks (2w pd2), 3-months (3m pd2), and 6-months post dose 2 (6m pd2); Boost, and 2-weeks post boost (2w pb). (**A**) Spike IgG antibody titers were 9-fold higher in individuals with prior COVID-19 infection than uninfected (Sero-Naïve) from 2w to 6m pd2. After Boost, titers increased but were not associated w/ prior infection. (**B** & **C**) Individuals with mild frailty compared to severe frailty had 5-fold higher spike and 11-fold higher RBD antibody titers from 2w to 6m pd2. After Boost, the impact of frailty status on antibody titers to spike and RBD was no longer significant. (**D**) Avidity of spike IgG antibody significantly decreased in all older adults over 6 months pd2 (p<0.001), but COVID-19 infection and frailty were not significantly associated factors. After the boost, antibody avidity improved with a trend towards significance in less frail individuals. P-values are included in the figure for the associations of clinical variables (eg. frailty) and titers.

Antibody responses were assessed quantitatively with titers and qualitatively with avidity, and the association of clinical factors with each participant’s antibody responses over the time course were evaluated using mixed-effects models [Figure 1]. Antibody titers after 2 vaccine doses, from peak response at 2-weeks through 6-months post second dose, were overall 9-fold higher for spike IgG antibody [Figure 1A] and 6-fold higher for RBD IgG antibody [data not shown] in individuals with prior COVID-19 infection compared to non-infected individuals (p < 0.001 and p = 0.06, respectively). When the impact of frailty status was evaluated, individuals with mild frailty compared to severe frailty had an overall 5-fold higher spike titer and 11-fold higher RBD antibody titers (p = 0.015 and p < 0.001, respectively) [Figure 1B–C]. This was not the result of biasing, as there was equal distribution of those with prior COVID-19 infection across frailty groups. Age and sex did not appear to be significantly associated with antibody titers in our limited study population [data not shown]. While there were differences in the absolute level of antibody from peak to trough (6-months), the degree and kinetics of antibody loss measured as percent change from peak to 6-months post second dose showed no significant differences by frailty or COVID-19 infection status.

Following the booster dose (comparing 6-months post second dose to 2-weeks post booster dose), all individuals had improved antibody titers (n = 8) [Figure 1A–C]. The impact of COVID-19 infection or frailty status on post-booster antibody titers to spike and RBD was no longer statistically significant (COVID-19 infection (p = 0.078 and p = 0.076, respectively) and frailty (p = 0.46 and p = 0.46, respectively)) [Figure 1A–C]. This suggests a ceiling effect on antibody quantity following multiple antigen exposures through vaccines and/or infection.

Assessment of antibody avidity revealed a significant decrease from peak response (2-weeks post second dose) through 6-months post second dose (p < 0.001), but COVID-19 infection and frailty were not significantly associated factors. Antibody avidity improved significantly after booster vaccine dose compared to the peak response. When evaluating the effects of frailty on this property, we found individuals with less frailty had higher avidity IC50 values with a trend towards significance (p = 0.060) compared to those with severe frailty, while prior COVID-19 infection lacked significance (p = 0.48) [Figure 1D].

### Cellular Responses

**Figure 2:**
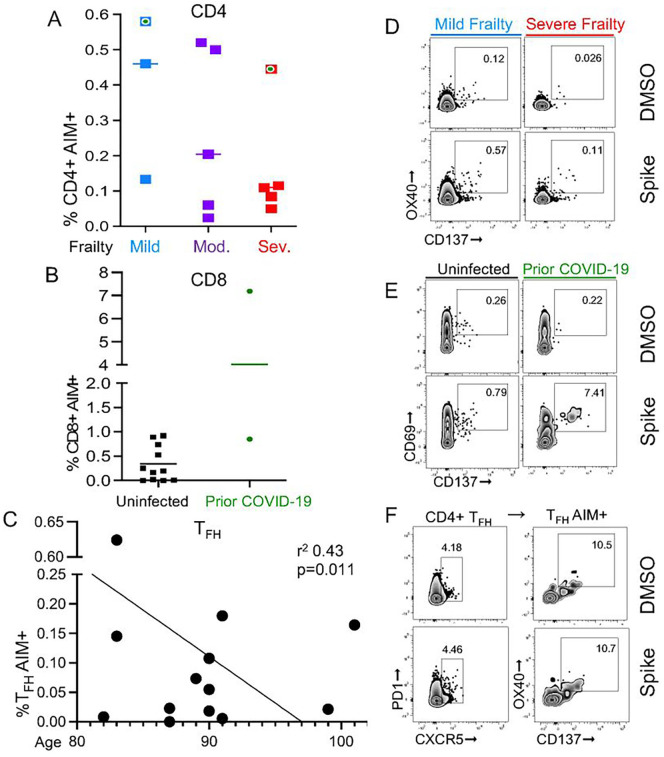
Frailty and Age Impact T Cell Responsiveness. The flow cytometry AIM assay was used to assess CD4+ and CD8+ T cell responsiveness after stimulation with pooled spike peptides (0.8ug/mL) or equimolar DMSO negative control for 24 hrs in PBMCs from (n=13) participants collected 2-weeks post vaccine dose 2. (**A**) Individuals with prior COVID-19, indicated by a green dot, (p=0.055) and mild frailty (p=0.13) had a trend towards increased responding AIM+CD4+ cells. (**B**) Prior COVID-19 infection was positively associated with AIM+CD8+ cells (p=0.0045). (**C**) Older aged individuals had decreased AIM+ T_FH_ cells (p=0.011). Representative flow plots with spike and DMSO negative control of (**D**) CD4+ AIM by mild (blue line) or severe (red line) frailty status, (**E**) CD8+ AIM by uninfected (black line) or prior COVID-19 (green line), and (**F**) CD4+ T_FH_ cells, defined by CD4+ CD4+CXCR5+PD1+ cells, and AIM with OX40+CD137+.

We analyzed spike-specific cellular responses in n = 13 participants at peak response (2-weeks post) to the second dose. For this study, PBMC were stimulated with peptide pools from the spike protein. Responding cells were determined by CD69 + CD137+ (CD8 + T cells) or OX40 + CD137+ (CD4 + T cells) [representative data are shown in Fig. 2D–2E, and gate selection is shown in Supplementary Fig. 1]. The total antibody response, calculated using the geometric mean of titers from baseline through 6-months post 2 vaccine doses, was positively associated with AIM + CD8 + and AIM + CD4 + cells from 2-weeks post (p = 0.022, r^2^ = 0.39 and p = 0.019, r^2^ = 0.41; respectively) [data not shown]. AIM + circulating T_FH_ cells, defined by CD4 + CXCR5 + PD1+, were not associated with antibody titers [data not shown].

Next, we analyzed clinical variables in combination with the immune phenotype of participants to identify a signature that could predict vaccine-specific T cell responses. Individuals with prior COVID-19 had the highest AIM + CD8 + cell response 2 weeks following the second dose (p = 0.0045) [Figure 2B]. Multiple predictors, including individuals with milder frailty in combination with fewer CD4 + terminally differentiated effector memory (TEMRA) cells and increased total T_FH_ cells had the highest AIM + CD4 + cell responses (p = 0.043, p = 0.023, and p = 0.069, respectively) [data not shown]. While older age was negatively correlated with AIM + T_FH_ cells (p = 0.011) [Figure 2C]. Finally, activated B cells (CD19 + CD69+) detected ex vivo were negatively associated with frailty, i.e. they were present at a 10% lower frequency for each increased degree on the CFS frailty scale, though this did not reach the cutoff for statistical significance (p = 0.075) [data not shown]. These results from analysis of cellular function inform the relationship between frailty status and age on vaccine-induced, spike-specific cellular responses.

**Figure 3:**
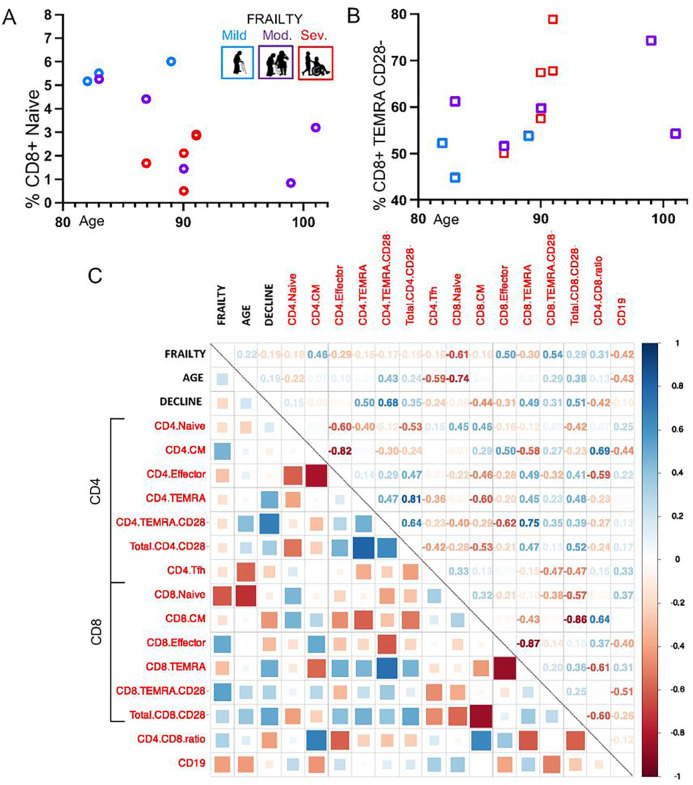
The Immune-Phenotype of Frailty and Old Age. The hallmarks of immune-senescence were compared in association with participant frailty status and age. (**A**) CD8+ naïve cells were decreased with higher frailty (p=0.019) and older age (p=0.002), and (**B**) CD8+ TEMRA CD28− cells were increased with severe frailty (p=0.0048), but not older age (p=0.5). (**C**) A heat map was used to represent correlation of clinical factors (frailty, age, and functional decline) with T cell memory populations: naïve, central memory (CM), effector, and terminally-differentiated effector memory (TEMRA), and CD28− was used as a marker of T cell aging. The strength of relationship was represented pictographically with boxes (blue is positive, red is negative) and numerically with correlation coefficients. Key results include frailty had the strongest positive correlation with CD8+ TEMRA CD28− cells (r=0.54) and negative correlation with CD8+ naive cells (r=−0.61). Age was negatively correlated with CD8+ naive cells (r=−0.74) and CD4+ T_FH_ cells (r=−0.59). Individuals with increased frailty over the study period, regardless of baseline characteristics, had strong correlations with CD4+ TEMRA CD28− cells (r=0.68)

Additionally, we characterized the immune landscape in our study cohort. T cell memory was described with naïve (CD45RA + CCR7+), central memory (CD45RA− CCR7+), effector memory (CD45RA− CCR7−), and terminally-differentiated effector memory TEMRA (CD45RA + CCR7−) populations,^42^ and the lack of CD28 co-receptor was used as a marker of T cell aging.^78^ Based on prior evidence of impaired influenza vaccine responses in older adults,^44,45^ CD8 + naive cells and CD8 + CD28− TEMRA cells were selected for association analyses with age and frailty. CD8 + naïve cells were decreased in individuals with a higher degree of frailty (p = 0.019) and older age (p = 0.002) [Figure 3A]. CD8 + TEMRA CD28− cells were increased with severe frailty (p = 0.0048), but not older age (p = 0.5) [Figure 3B].

Furthermore, we conducted analysis across the T cell memory populations to explore markers of immune-senescence correlated with frailty, age, and functional decline. In validation of the preceding univariate analysis results, participant frailty status had the strongest positive correlation with CD8 + CD28− TEMRA cells (r = 0.54) and negative correlation with CD8 + naive cells (r= −0.61) [Figure 3C]. Older age had strong negative correlations with CD8 + naive cells (r= −0.74) and CD4 + T_FH_ cells (r= −0.59) [Figure 3C]. Interestingly, individuals who experienced functional decline during the study period (n = 7) were found to have positive correlations with CD4 + CD28− TEMRA cells (r = 0.68) regardless of their baseline frailty status [Figure 3C]. These findings provide new insights into adaptive immune system dysregulation, with comparisons and contrasts between frailty and age.

## Discussion

In this small cohort of older adults living in a retirement community, we characterized the adaptive immune responses of frail older adults to the Moderna COVID-19 mRNA vaccine. Overall, the frail older study participants generated high levels of antibody following vaccination that reached similar levels following a booster vaccine. As expected, individuals with prior COVID-19 infection had higher baseline antibodies and reached a higher level at peak (2-weeks post 2nd dose).

Higher frailty was associated with decreased antibody quantity after the first 2 vaccine doses. Age had less of an impact on antibody titers than frailty within our population comprised of very old individuals. This is in agreement with studies of the Zoster vaccine.^11^ We found a parallel pattern of waning antibody regardless of frailty or prior infection. Importantly, the booster vaccination overcame the effects of COVID-19 infection and frailty on antibody quantity. This suggests maximal generation of antibody can be reached with appropriate boosting even in frail older adults.

Antibody avidity waned significantly over the 6 months following the second dose for all older adult participants. This is in stark contrast to findings in younger healthy adults where anti-spike IgG avidity increased from 2 weeks to 6 months following the second vaccine dose, which was accompanied by persistent GC reactions and somatic hypermutation in responding B cells.^63^ The substantial decrease in avidity observed in our study is consistent with poor or attenuated germinal center reactions in older individuals resulting in the failure to generate higher avidity antibody secreting cells at later time points following vaccination.

Remarkably, anti-spike IgG avidity increased rapidly after the boost. The increase in avidity early after the boost suggests either generation of higher avidity memory B cells that originated from the initial doses of vaccine or rapid affinity maturation following the boost. Older age has been associated with impaired germinal center responses and vaccine-specific antibody generation following influenza vaccination, but surprisingly older adults had similar avidity compared to younger adults, indicating effects from prior antigen exposure.^39,79^ As SARS-CoV-2 is a *de novo* virus, B cell and antibody responses in older adults following vaccination represents an important area for future study. As with antibody quantity, frailty may play a role in this process, as we noted a trend towards higher avidity antibodies after the booster vaccine in individuals with less frailty. We postulate individuals with high levels of frailty may have further decreases in germinal center responses implicating immune-senescent changes in B cells and/or CD4 + T_FH_ cells, which are critical for long-lived high avidity responses.

To gain insights into cellular responsiveness, we described a relationship between spike-specific activation of CD4 + and CD8 + T cells with total antibody response after 2 vaccine doses. Prior studies have described impaired influenza vaccine antibody production in older adults was correlated with increased frequencies of CD8 + CD28− T cells and decreased number of T_FH_ cells after vaccination.^43–45^ In our participants, spike-specific CD4 + and CD8 + T cell responses correlated with antibody responses. We found frailty status in conjunction with CD4 + TEMRA cells was inversely associated with spike-specific CD4 + T cell function. The repertoire of TEMRA T cells is recognized as a feature of the aging immune system with detrimental inflammatory responses.^42,49^ This indicates frailty, as a measure of biological age, in interaction with an individual’s underlying immune phenotype influences their CD4 + T cell vaccine responses. In contrast to frailty, older age alone was inversely associated with spike-specific T_FH_ function. The alterations in the T_FH_ cell population in older adults may be key drivers of impaired germinal center responses and decreased avidity, as described above. Our findings are among the first to describe the independent impacts of frailty and age on cellular vaccine responses.

We further explored the association of cellular immune-senescence with clinical factors including frailty and age. We found decreased naïve CD8 + cells in individuals with a higher degree of frailty and older age. Yet, CD8 + CD28− TEMRA cells were only significantly associated with higher frailty. Not surprisingly, we found some individuals experienced declining health and functional status over the study period. Regardless of their degree of frailty, these individuals had increased CD4 + CD28− TEMRA cells at a time of immune activation (2 weeks post second dose). Chronic stimulation of an aged immune system is thought to result in CD8 + CD28− TEMRA cells, which are associated with decreased responsiveness.^78^ In contrast, the absence of CD28 on CD4 + TEMRA cells is usually transient and activation-induced, and the presence of CD4 + CD28− cells have been associated with vascular inflammation and stroke.^80–82^ Thus, immune-senescence in CD8 + T cells may be sequelae of chronic inflammation resulting in biological frailty, and dysfunction in the CD4 + subset may be an indicator for actively declining health.

### Strengths and Weaknesses

Using a study design including sample collections, assay selection, and timeframe modeled after the phase 3 trial results published from the Moderna vaccine group, we addressed a range of questions in the field of aging immunology.^49,55^ We investigated the antibody response and T-cell functionality in a vulnerable population of frail older adults to a novel vaccine. While vaccine immunology publications have incorporated the chronological age of older adults, there has been little focus on the gero-science concepts of biological age and frailty. Reliable measures of frailty including the CFS and have been described in a limited number of COVID-19 vaccine studies in the nursing home setting.^25,26,57^ A single-site nursing home study in France had little variability in frailty status, which limited their ability to detect the association of frailty with antibody and cellular responses.^25^ Interestingly, a study from Poland with mixed frailty status of participants described little effect of frailty on antibody responses to the Pfizer mRNA vaccine, though their measurements did not include assessment past 2-weeks post second vaccine dose.^26^ Finally, a nursing home study from Ireland described frailty in association with decreased antibody responses at 6 months post vaccination, but they did not report cellular vaccine responses. Our study increases the understanding of immune responses to COVID-19 mRNA vaccination in older adults, demonstrating frailty and age in older adults are independently associated with impaired responsiveness and immune-senescence. Understanding the vaccine response of the aged immune system has significant implications for vaccination strategies in this vulnerable population, including guidance on booster doses.

The main limitations of our study are the small sample size and loss of study participants at the booster vaccine sample collection. While other COVID-19 vaccine studies in nursing homes have used younger health care workers as a control population, the younger and less frail participants in our study cohort served as an internal control. In addition, the restricted range in our population’s scoring on the Clinical Frailty Scale from mildly frail to severely frail, did not capture the full range of clinical and functional health status. These aspects of our study design introduce limitations on the statistical power and external validity of our findings. We used mixed effects linear regression to measure individual participant antibody responses over time, and though a valid statistical method, it is not common in immunological studies. Our hope is to perform longitudinal profiling in a larger sample of older adults. The assays were selected for their rigorous performance, but we did not include cytokine assays for quantitation of cellular responses or analysis of responses to SARS-CoV-2 variants including Delta and Omicron. We did not study cellular response after the booster dose, but T cell activation has been reported to remain stable between the second and the first booster COVID-19 mRNA dose in older individuals.^72,83^

## Conclusion

Our findings reveal new insights into the immune response to COVID-19 vaccination in older adults and the interplay of age and frailty on the immune system. Vaccine responses in frail individuals were impaired across the adaptive immune system, spanning decreased antibody titers and avidity as well as impaired cellular function accompanied by a high burden of immune-senescent T cells. Importantly, the booster dose improved antibody titers in all older adults, mitigating the effects of frailty or prior COVID-19 infection. We also found significantly lower avidity antibody at later times (6-months) following the second vaccine dose in all older adults, in contrast to findings in younger healthy adults.^63^ A boost promoted increases in antibody avidity that surpassed the second vaccine dose, suggesting decreased competence of germinal center responses to initial antigen exposure and predilection for memory B cell generation in older adults.

We found antigen-specific CD4 + cell responses were independently impacted by frailty and age. Further, accumulation of CD8 + CD28− TEMRA cells was associated with frailty, while CD4 + CD28-TEMRA cells served as an indicator of declining health regardless of baseline frailty status. These data support a model where the aging immune system contributes to frailty through excessive inflammatory responses.^49,73^ Together our data show chronological age and frailty, as a measure of biological age, are independently associated with alterations in the aging immune system, while opening the door to important therapeutic interventions including vaccine booster recommendations.

## Supplementary Material

Supplement 1

Supplement 2
