# Supplementary material for: Frailty and Age Impact Immune Responses to Moderna COVID-19 mRNA Vaccine
Source: Res Sq. 2022 Aug 1:rs.3.rs-1883093. Preprint. [Version 1] doi: 10.21203/rs.3.rs-1883093/v1 (PMC9387536; doi:10.21203/rs.3.rs-1883093/v1)
Supplement: Supplement 1 [file FVTable1.pdf]

| Table 1. Retirement Community Cohort | |
|---|---|
| **Living Setting*** | |
| Nursing Home | 5 (33%) |
| Assisted Living Facility | 10 (67%) |
| **Prior COVID-19 infection*** | 3 (20%) |
| **Sex, Female*** | 12 (80%) |
| **Race, White (non-Hispanic)*** | 15 (100%) |
| **Age, median (min, max)** | 90 years (62 – 101) |
| **Age Histogram** | 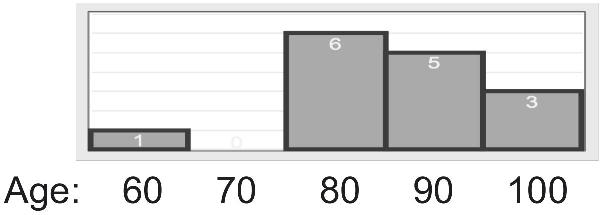 |
| **Clinical Frailty Scale (CFS)*** | |
| Mild Frailty | 3 (20%) |
| Moderate Frailty | 7 (47%) |
| Severe Frailty | 5 (33%) |

**Sample Collections***

| Baseline Dose 1 | Dose 2 (28 days) | 2 weeks p. Dose 2 | 3 months p. Dose 2 | 6 months p. Dose 2 | 2 weeks p. Boost |
|---|---|---|---|---|---|
| 9 (60%) | 15 (100%) | 14 (93%) | 13 (87%) | 14 (93%) | 8 (53%) |

*N (%)
