# Supplementary material for: Frailty and Age Impact Immune Responses to Moderna COVID-19 mRNA Vaccine
Source: Res Sq. 2022 Aug 1:rs.3.rs-1883093. Preprint. [Version 1] doi: 10.21203/rs.3.rs-1883093/v1 (PMC9387536; doi:10.21203/rs.3.rs-1883093/v1)
Supplement: Supplement 2 [file SupplementFrailVaccineResponses.pdf]

**Supplementary Figure 1.**
Sample Gating Strategy

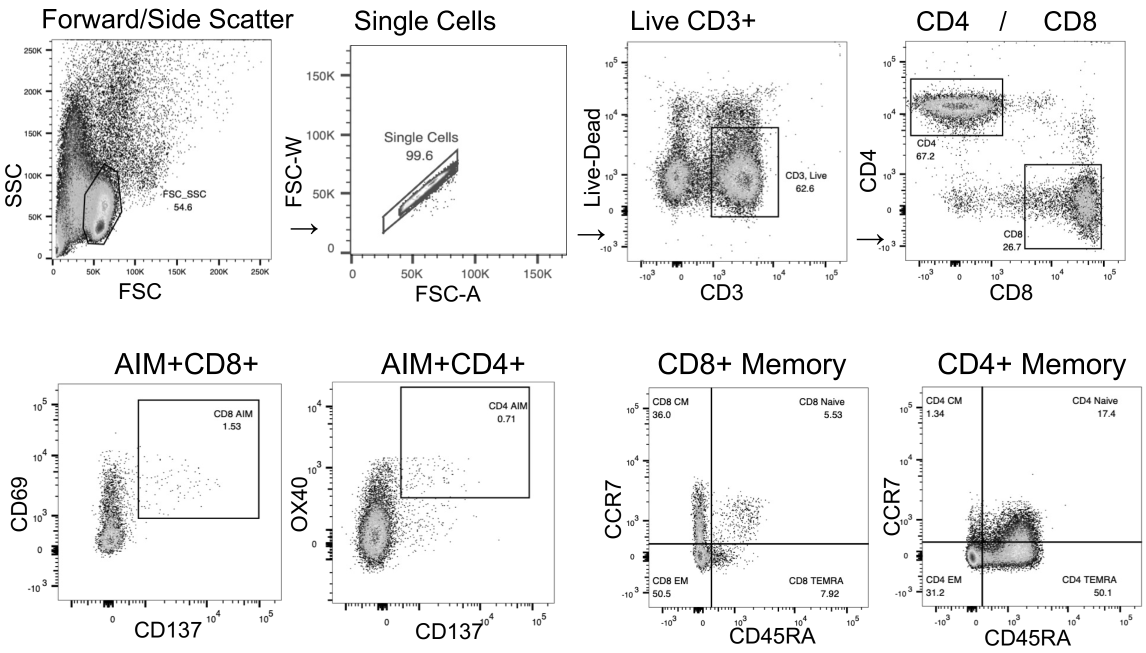


**Supplementary Table 1.**

| **Flow Cytometer Antibody Panel** | | |
|---|---|---|
| **Marker** | **Fluor** | **Clone** |
| Live/Dead Zombie Aqua | BV510 | |
| CD3 | AF700 | SK7 |
| CD4 | BV605 | SK3 |
| CD8 | BV650 | SK1 |
| CD19 | PerCP/Cy5.5 | HIB19 |
| CCR7 | PE/Cy7 | G043H7 |
| CD45RA | ACP/Cy7 | HI100 |
| CXCR5 | AF647 | J252D4 |
| CD28 | BV711 | CD28.2 |
| CD69 | KB520 | FN50 |
| PD1 | PE | A17188B |
| CD137 | PE/Dazzle 594 | 4B4-1 |
| OX40 | BV421 | ACT35 |
